# Supplementary material for: Reaching the last mile with ivermectin mass drug administration against onchocerciasis: The case of Kwanware-Ottou persistent transmission focus in the Wenchi health district of Ghana
Source: PLoS Negl Trop Dis. 2026 Feb 5;20(2):e0013958. doi: 10.1371/journal.pntd.0013958 (PMC12900439; doi:10.1371/journal.pntd.0013958)
Supplement: S1 Text — (DOCX) [file pntd.0013958.s001.docx]

Appendix 1. Census Questionnaire

[1. Which District/Municipal are you in? /data/district](https://www.commcarehq.org/a/nigeria-1/apps/view/d5b98f27d7ef43429a79f420f88c70af/form/adab5ea8b48f4f6abf5ed1c5594dc673/source/#form/district) ^required^

- Tain tain
- Wenchi wenchi
- Banda banda

[2. Which Sub-district are you in? /data/Subdistrict](https://www.commcarehq.org/a/nigeria-1/apps/view/d5b98f27d7ef43429a79f420f88c70af/form/adab5ea8b48f4f6abf5ed1c5594dc673/source/#form/Subdistrict) ^required^

- Subinso subinso
- Nsawkaw nsawkaw
- Other other

[3. if other specify /data/oth_subdistrict](https://www.commcarehq.org/a/nigeria-1/apps/view/d5b98f27d7ef43429a79f420f88c70af/form/adab5ea8b48f4f6abf5ed1c5594dc673/source/#form/oth_subdistrict) ^required^

[4. Select community /data/community](https://www.commcarehq.org/a/nigeria-1/apps/view/d5b98f27d7ef43429a79f420f88c70af/form/adab5ea8b48f4f6abf5ed1c5594dc673/source/#form/community) ^required^

- Abotereye aboteyere
- Adamukura adamukura
- Branam branam
- Krachikrom krachikrom
- Kwanware kwanware
- Ottou ottou
- Subinso subinso
- Tainano tainano
- Others oth_comm

[5. If others, specify /data/oth_comm_specified](https://www.commcarehq.org/a/nigeria-1/apps/view/d5b98f27d7ef43429a79f420f88c70af/form/adab5ea8b48f4f6abf5ed1c5594dc673/source/#form/oth_comm_specified) ^required^

[6. HH CONSENT: Hello. I am here on behalf of the GHS/NTD programme. We are surveying houses in your community about the most recent community drug distribution. We would like to know if the members of your house took the drugs. If you wish to participate in the survey, tell us and we will note your answer. If you agree, the survey will only take a few minutes. Taking part in the house survey will not benefit you directly, but the results will help the Ghana Health Service NTD programme improve upon ivermectin/Mectizan distribution against onchocerciasis. It is your choice to take part, or not to take part, in this survey. You may refuse without penalty. Would you like to take part in our survey? /data/hh_consent](https://www.commcarehq.org/a/nigeria-1/apps/view/d5b98f27d7ef43429a79f420f88c70af/form/adab5ea8b48f4f6abf5ed1c5594dc673/source/#form/hh_consent) ^required^

- Given 1
- Household Absent 2
- Refused 0

[7. Name of nearest health facility /data/nearest_hf](https://www.commcarehq.org/a/nigeria-1/apps/view/d5b98f27d7ef43429a79f420f88c70af/form/adab5ea8b48f4f6abf5ed1c5594dc673/source/#form/nearest_hf) ^required^

[8. Head of household name /data/hh_name](https://www.commcarehq.org/a/nigeria-1/apps/view/d5b98f27d7ef43429a79f420f88c70af/form/adab5ea8b48f4f6abf5ed1c5594dc673/source/#form/hh_name) ^required^

[9. House Number /data/HH_number](https://www.commcarehq.org/a/nigeria-1/apps/view/d5b98f27d7ef43429a79f420f88c70af/form/adab5ea8b48f4f6abf5ed1c5594dc673/source/#form/HH_number) ^required^

[10. GPS location /data/gps](https://www.commcarehq.org/a/nigeria-1/apps/view/d5b98f27d7ef43429a79f420f88c70af/form/adab5ea8b48f4f6abf5ed1c5594dc673/source/#form/gps) ^required^

[11. How many individuals live in your household? /data/num_reside](https://www.commcarehq.org/a/nigeria-1/apps/view/d5b98f27d7ef43429a79f420f88c70af/form/adab5ea8b48f4f6abf5ed1c5594dc673/source/#form/num_reside) ^required^

[Individual Information /data/group_indv](https://www.commcarehq.org/a/nigeria-1/apps/view/d5b98f27d7ef43429a79f420f88c70af/form/adab5ea8b48f4f6abf5ed1c5594dc673/source/#form/group_indv)

[14. INDIVIDUAL CONSENT: Hello. I am here on behalf of the GHS/NTD programme. We are surveying houses in your community about the most recent community drug distribution. We would like to know if you took the drugs. If you wish to participate in the survey, tell us and we will note your answer. If you agree, the survey will only take a few minutes. Taking part in the house survey will not benefit you directly, but the results will help the Ghana Health Service NTD programme improve upon ivermectin/Mectizan distribution against onchocerciasis. It is your choice to take part, or not to take part, in this survey. You may refuse without penalty. Would you like to take part in our survey? /data/group_indv/indiv_consent](https://www.commcarehq.org/a/nigeria-1/apps/view/d5b98f27d7ef43429a79f420f88c70af/form/adab5ea8b48f4f6abf5ed1c5594dc673/source/#form/group_indv/indiv_consent) ^required^

- Given 1
- Individual absent but will answer on his/her behalf 2
- Individual absent and I prefer not to answer on his/her behalf 3
- Refused 0

[15. Take a picture of the consent form /data/group_indv/consent_pic](https://www.commcarehq.org/a/nigeria-1/apps/view/d5b98f27d7ef43429a79f420f88c70af/form/adab5ea8b48f4f6abf5ed1c5594dc673/source/#form/group_indv/consent_pic)

[Consenting individual /data/group_indv/indiv_consented](https://www.commcarehq.org/a/nigeria-1/apps/view/d5b98f27d7ef43429a79f420f88c70af/form/adab5ea8b48f4f6abf5ed1c5594dc673/source/#form/group_indv/indiv_consented)

[19. Person Responding? /data/group_indv/indiv_consented/responder](https://www.commcarehq.org/a/nigeria-1/apps/view/d5b98f27d7ef43429a79f420f88c70af/form/adab5ea8b48f4f6abf5ed1c5594dc673/source/#form/group_indv/indiv_consented/responder) ^required^

- Themselves 1
- Someone on their behalf as absent 2
- Someone on their behalf as too young 3

[16. Name of individual /data/group_indv/indiv_consented/name_resp](https://www.commcarehq.org/a/nigeria-1/apps/view/d5b98f27d7ef43429a79f420f88c70af/form/adab5ea8b48f4f6abf5ed1c5594dc673/source/#form/group_indv/indiv_consented/name_resp) ^required^

[18. Sex /data/group_indv/indiv_consented/sex](https://www.commcarehq.org/a/nigeria-1/apps/view/d5b98f27d7ef43429a79f420f88c70af/form/adab5ea8b48f4f6abf5ed1c5594dc673/source/#form/group_indv/indiv_consented/sex) ^required^

- Male 1
- Female 2

[17. Age (years) /data/group_indv/indiv_consented/age](https://www.commcarehq.org/a/nigeria-1/apps/view/d5b98f27d7ef43429a79f420f88c70af/form/adab5ea8b48f4f6abf5ed1c5594dc673/source/#form/group_indv/indiv_consented/age) ^required^

[22. Is this individual recorded in the just ended (2024) MDA register? /data/group_indv/indiv_consented/MDA_census](https://www.commcarehq.org/a/nigeria-1/apps/view/d5b98f27d7ef43429a79f420f88c70af/form/adab5ea8b48f4f6abf5ed1c5594dc673/source/#form/group_indv/indiv_consented/MDA_census) ^required^

- Yes 1
- No 0
- Do not know/Can not remember 99

[36. Were you tested for onchocerciasis in 2019 /data/group_indv/indiv_consented/snip_19_or_20](https://www.commcarehq.org/a/nigeria-1/apps/view/d5b98f27d7ef43429a79f420f88c70af/form/adab5ea8b48f4f6abf5ed1c5594dc673/source/#form/group_indv/indiv_consented/snip_19_or_20) ^required^

- yes yes
- no no
- Do not remember 99

[What was the result of your test? /data/group_indv/indiv_consented/snip_result](https://www.commcarehq.org/a/nigeria-1/apps/view/d5b98f27d7ef43429a79f420f88c70af/form/adab5ea8b48f4f6abf5ed1c5594dc673/source/#form/group_indv/indiv_consented/snip_result) ^required^

- positive positive
- negative negative
- Do not remember/ Do not remember 99

[20. What is your ethnicity/tribe? /data/group_indv/indiv_consented/ethnicity](https://www.commcarehq.org/a/nigeria-1/apps/view/d5b98f27d7ef43429a79f420f88c70af/form/adab5ea8b48f4f6abf5ed1c5594dc673/source/#form/group_indv/indiv_consented/ethnicity) ^required^

- Akan 1
- Ewe 2
- Dagomba 3
- Guan 4
- Other (specify) 5

[21. If others, specify /data/group_indv/indiv_consented/oth_ethnicity](https://www.commcarehq.org/a/nigeria-1/apps/view/d5b98f27d7ef43429a79f420f88c70af/form/adab5ea8b48f4f6abf5ed1c5594dc673/source/#form/group_indv/indiv_consented/oth_ethnicity) ^required^

[28. What is the highest level of education you have attained? /data/group_indv/indiv_consented/education](https://www.commcarehq.org/a/nigeria-1/apps/view/d5b98f27d7ef43429a79f420f88c70af/form/adab5ea8b48f4f6abf5ed1c5594dc673/source/#form/group_indv/indiv_consented/education) ^required^

- Have never attended School 0
- Attended but did not finish a level 1
- Not attending; primary education completed 2
- Not attending; secondary education completed 3
- Not attending; tertiary education completed 4
- Currently attending school primary school 5
- Currently attending school secondary school 6
- Currently attending school tertiary school 7
- Currently not attending but a level of arabic/Islamiyah school/education 8
- Currently attending Arabic school 9

[. CES /data/group_indv/indiv_consented/ces](https://www.commcarehq.org/a/nigeria-1/apps/view/d5b98f27d7ef43429a79f420f88c70af/form/adab5ea8b48f4f6abf5ed1c5594dc673/source/#form/group_indv/indiv_consented/ces)

[23. Is this place your permanent home? /data/group_indv/indiv_consented/ces/residence](https://www.commcarehq.org/a/nigeria-1/apps/view/d5b98f27d7ef43429a79f420f88c70af/form/adab5ea8b48f4f6abf5ed1c5594dc673/source/#form/group_indv/indiv_consented/ces/residence) ^required^

- yes yes
- no no

[24. Where is your permanent home? /data/group_indv/indiv_consented/ces/resident_name](https://www.commcarehq.org/a/nigeria-1/apps/view/d5b98f27d7ef43429a79f420f88c70af/form/adab5ea8b48f4f6abf5ed1c5594dc673/source/#form/group_indv/indiv_consented/ces/resident_name) ^required^

[25. How long have you been permanently here? /data/group_indv/indiv_consented/ces/permanent_time](https://www.commcarehq.org/a/nigeria-1/apps/view/d5b98f27d7ef43429a79f420f88c70af/form/adab5ea8b48f4f6abf5ed1c5594dc673/source/#form/group_indv/indiv_consented/ces/permanent_time) ^required^

- < 3 months 1
- 6-12 months 2
- 1-2 years 3
- 3-5 years 4
- 5-10 years 5
- >10 years 6

[26. How long have you stayed in the community this time around? /data/group_indv/indiv_consented/ces/visit_duration](https://www.commcarehq.org/a/nigeria-1/apps/view/d5b98f27d7ef43429a79f420f88c70af/form/adab5ea8b48f4f6abf5ed1c5594dc673/source/#form/group_indv/indiv_consented/ces/visit_duration) ^required^

- < 3 months 1
- 3-6 month month
- 6-12 months 2
- 1-2 years 3
- 3-5 years 4
- 5-10 years 5
- >10 years 6

[27. How long have you been visiting this community? /data/group_indv/indiv_consented/ces/length_comm](https://www.commcarehq.org/a/nigeria-1/apps/view/d5b98f27d7ef43429a79f420f88c70af/form/adab5ea8b48f4f6abf5ed1c5594dc673/source/#form/group_indv/indiv_consented/ces/length_comm) ^required^

- year 1
- 1-2 years 2
- 3-5 years 3
- 5-10 years 4
- >10 years 5

[35. Have you been out of the community during the last 12 months? /data/group_indv/indiv_consented/ces/outside_comm](https://www.commcarehq.org/a/nigeria-1/apps/view/d5b98f27d7ef43429a79f420f88c70af/form/adab5ea8b48f4f6abf5ed1c5594dc673/source/#form/group_indv/indiv_consented/ces/outside_comm) ^required^

- yes yes
- no no

[37. How many times have you move outside this community (for one month or more) in the last 12 months /data/group_indv/indiv_consented/ces/number_months_outside](https://www.commcarehq.org/a/nigeria-1/apps/view/d5b98f27d7ef43429a79f420f88c70af/form/adab5ea8b48f4f6abf5ed1c5594dc673/source/#form/group_indv/indiv_consented/ces/number_months_outside) ^required^

[I am now going to ask about your movement over the past recent 12 months /data/group_indv/indiv_consented/ces/question6](https://www.commcarehq.org/a/nigeria-1/apps/view/d5b98f27d7ef43429a79f420f88c70af/form/adab5ea8b48f4f6abf5ed1c5594dc673/source/#form/group_indv/indiv_consented/ces/question6)

[40. Which month/s were you out of the community? /data/group_indv/indiv_consented/ces/question6/outside_month](https://www.commcarehq.org/a/nigeria-1/apps/view/d5b98f27d7ef43429a79f420f88c70af/form/adab5ea8b48f4f6abf5ed1c5594dc673/source/#form/group_indv/indiv_consented/ces/question6/outside_month) ^required^

- January january
- February february
- March march
- April april
- May may
- June june
- July july
- August august
- September september
- October october
- November november
- December december

[/data/group_indv/indiv_consented/ces/question6/question1](https://www.commcarehq.org/a/nigeria-1/apps/view/d5b98f27d7ef43429a79f420f88c70af/form/adab5ea8b48f4f6abf5ed1c5594dc673/source/#form/group_indv/indiv_consented/ces/question6/question1)

[41. Where were you? /data/group_indv/indiv_consented/ces/question6/question1/inside_this_district](https://www.commcarehq.org/a/nigeria-1/apps/view/d5b98f27d7ef43429a79f420f88c70af/form/adab5ea8b48f4f6abf5ed1c5594dc673/source/#form/group_indv/indiv_consented/ces/question6/question1/inside_this_district) ^required^

- Outside this district choice12
- Inside this district inside_the_district

[42. Which community were you in? /data/group_indv/indiv_consented/ces/question6/question1/state_the_community](https://www.commcarehq.org/a/nigeria-1/apps/view/d5b98f27d7ef43429a79f420f88c70af/form/adab5ea8b48f4f6abf5ed1c5594dc673/source/#form/group_indv/indiv_consented/ces/question6/question1/state_the_community) ^required^

[Did you received ivermectin/Mectizan in this community? /data/group_indv/indiv_consented/ces/question6/question1/outside_comm_ivm](https://www.commcarehq.org/a/nigeria-1/apps/view/d5b98f27d7ef43429a79f420f88c70af/form/adab5ea8b48f4f6abf5ed1c5594dc673/source/#form/group_indv/indiv_consented/ces/question6/question1/outside_comm_ivm) ^required^

- yes yes
- no no
- Do not do_not

[29. What is your main occupation? /data/group_indv/indiv_consented/ces/main_occupation](https://www.commcarehq.org/a/nigeria-1/apps/view/d5b98f27d7ef43429a79f420f88c70af/form/adab5ea8b48f4f6abf5ed1c5594dc673/source/#form/group_indv/indiv_consented/ces/main_occupation) ^required^

- Farming 1
- Student/going to school 2
- Fishing 3
- Hunting 4
- Mining 5
- Sale services/business 6
- Office worker/teacher/Civil servants 7
- Others 8

[30. If other, specify /data/group_indv/indiv_consented/ces/oth_main_occupation](https://www.commcarehq.org/a/nigeria-1/apps/view/d5b98f27d7ef43429a79f420f88c70af/form/adab5ea8b48f4f6abf5ed1c5594dc673/source/#form/group_indv/indiv_consented/ces/oth_main_occupation) ^required^

[31. If mining, specify /data/group_indv/indiv_consented/ces/main_occup_mining_type](https://www.commcarehq.org/a/nigeria-1/apps/view/d5b98f27d7ef43429a79f420f88c70af/form/adab5ea8b48f4f6abf5ed1c5594dc673/source/#form/group_indv/indiv_consented/ces/main_occup_mining_type) ^required^

- Artisanal 1
- Mining with a company 2

[32. What is your other occupation? /data/group_indv/indiv_consented/ces/oth_occupation](https://www.commcarehq.org/a/nigeria-1/apps/view/d5b98f27d7ef43429a79f420f88c70af/form/adab5ea8b48f4f6abf5ed1c5594dc673/source/#form/group_indv/indiv_consented/ces/oth_occupation) ^required^

- Farming 1
- Student/going to school 2
- Fishing 3
- Hunting 4
- Mining 5
- Sale services/business 6
- Office worker/teacher/Civil servants 7
- Others 8
- None none

[33. If other, specify /data/group_indv/indiv_consented/ces/oth_occupation_others](https://www.commcarehq.org/a/nigeria-1/apps/view/d5b98f27d7ef43429a79f420f88c70af/form/adab5ea8b48f4f6abf5ed1c5594dc673/source/#form/group_indv/indiv_consented/ces/oth_occupation_others) ^required^

[34. If mining, specify /data/group_indv/indiv_consented/ces/oth_occupation_mining_type](https://www.commcarehq.org/a/nigeria-1/apps/view/d5b98f27d7ef43429a79f420f88c70af/form/adab5ea8b48f4f6abf5ed1c5594dc673/source/#form/group_indv/indiv_consented/ces/oth_occupation_mining_type) ^required^

- Artisanal 1
- Mining with a company 2

[43. Have you had any of the following: Leopard skin, Skin thickening, Skin itches, Blurred vision, Blindness? /data/group_indv/indiv_consented/ces/Oncho_morbidity](https://www.commcarehq.org/a/nigeria-1/apps/view/d5b98f27d7ef43429a79f420f88c70af/form/adab5ea8b48f4f6abf5ed1c5594dc673/source/#form/group_indv/indiv_consented/ces/Oncho_morbidity) ^required^

- yes yes
- no 0

[44. If yes, which one? /data/group_indv/indiv_consented/ces/oncho_morb_type](https://www.commcarehq.org/a/nigeria-1/apps/view/d5b98f27d7ef43429a79f420f88c70af/form/adab5ea8b48f4f6abf5ed1c5594dc673/source/#form/group_indv/indiv_consented/ces/oncho_morb_type) ^required^

- Visual impairment visual_impairment
- Skin thickening skin_thickening
- Leopard skin leopard_skin
- Blurred vision blurred_vision

[45. Were you OFFERED drugs for Oncho during the recent ivermectin MDA round in 2024? /data/group_indv/indiv_consented/ces/offered_ov](https://www.commcarehq.org/a/nigeria-1/apps/view/d5b98f27d7ef43429a79f420f88c70af/form/adab5ea8b48f4f6abf5ed1c5594dc673/source/#form/group_indv/indiv_consented/ces/offered_ov) ^required^

- Yes 1
- No 0
- Do not remember 9

[46. Did a CDD for the Oncho MDA programme visit your household during the last campaign? /data/group_indv/indiv_consented/ces/cdd_visited_ov](https://www.commcarehq.org/a/nigeria-1/apps/view/d5b98f27d7ef43429a79f420f88c70af/form/adab5ea8b48f4f6abf5ed1c5594dc673/source/#form/group_indv/indiv_consented/ces/cdd_visited_ov) ^required^

- Yes 1
- No 0
- Do not know / remember 9

[47. Were you present during the ivermectin /Mectizan MDA campaign? /data/group_indv/indiv_consented/ces/present_ov](https://www.commcarehq.org/a/nigeria-1/apps/view/d5b98f27d7ef43429a79f420f88c70af/form/adab5ea8b48f4f6abf5ed1c5594dc673/source/#form/group_indv/indiv_consented/ces/present_ov) ^required^

- Yes, was present during the ivermectin MDA campaign 1
- No, was absent during the ivermectin MDA campaign 0
- Do not Remember 9

[48. Since you were absent, where were you during the last ivermectin MDA campaign? /data/group_indv/indiv_consented/ces/absent_loc_ov](https://www.commcarehq.org/a/nigeria-1/apps/view/d5b98f27d7ef43429a79f420f88c70af/form/adab5ea8b48f4f6abf5ed1c5594dc673/source/#form/group_indv/indiv_consented/ces/absent_loc_ov) ^required^

- At school during the MDA visit 1
- At boarding school in another community 2
- At work during the MDA visit 3
- Travelling outside the community 4
- Visitor, normally reside in another community 5
- Other Specify 6
- Do not remember 9

[49. Other - Specify /data/group_indv/indiv_consented/ces/specify_absent_location](https://www.commcarehq.org/a/nigeria-1/apps/view/d5b98f27d7ef43429a79f420f88c70af/form/adab5ea8b48f4f6abf5ed1c5594dc673/source/#form/group_indv/indiv_consented/ces/specify_absent_location) ^required^

[50. Did you SWALLOW the drugs for Oncho given to you in the recent ivmerctin MDA round? /data/group_indv/indiv_consented/ces/swallow_ov](https://www.commcarehq.org/a/nigeria-1/apps/view/d5b98f27d7ef43429a79f420f88c70af/form/adab5ea8b48f4f6abf5ed1c5594dc673/source/#form/group_indv/indiv_consented/ces/swallow_ov) ^required^

- Yes 1
- Did not swallow 0
- Do not remember 9

[51. Why did you not swallow the drugs for Oncho /data/group_indv/indiv_consented/ces/notswallow_ovlf](https://www.commcarehq.org/a/nigeria-1/apps/view/d5b98f27d7ef43429a79f420f88c70af/form/adab5ea8b48f4f6abf5ed1c5594dc673/source/#form/group_indv/indiv_consented/ces/notswallow_ovlf) ^required^

- Did not know/trust the CDD 1
- Community Drug Distributor did not come 2
- Fear of side effects 3
- Is healthy 4
- Was sick / taking another medication during Ivermectin MDA 5
- Medicine does not work 6
- Too old 7
- Underage 8
- Tired of taking drugs 9
- Pregnant or breast-feeding 10
- Was absent 11
- Did not hear about the campaign 12
- Other (please specify) 99

[52. Other reason for not swallowing, please specify /data/group_indv/indiv_consented/ces/notswallowov_other](https://www.commcarehq.org/a/nigeria-1/apps/view/d5b98f27d7ef43429a79f420f88c70af/form/adab5ea8b48f4f6abf5ed1c5594dc673/source/#form/group_indv/indiv_consented/ces/notswallowov_other) ^required^

[54. Did you feel any side effects after swallowing the medication? /data/group_indv/indiv_consented/ces/side_effects_oncho](https://www.commcarehq.org/a/nigeria-1/apps/view/d5b98f27d7ef43429a79f420f88c70af/form/adab5ea8b48f4f6abf5ed1c5594dc673/source/#form/group_indv/indiv_consented/ces/side_effects_oncho) ^required^

- Yes 1
- No 2
- Don't know 9

[55. Please state what side effects you experienced after swallowing the drugs. /data/group_indv/indiv_consented/ces/side_effects_oncho_type](https://www.commcarehq.org/a/nigeria-1/apps/view/d5b98f27d7ef43429a79f420f88c70af/form/adab5ea8b48f4f6abf5ed1c5594dc673/source/#form/group_indv/indiv_consented/ces/side_effects_oncho_type) ^required^

- Vomiting 1
- Diarrhea 2
- Stomach ache - no vomiting 3
- Headache 4
- Fainting 5
- Others others

[If other side effects, specify /data/group_indv/indiv_consented/ces/oth_side_effect](https://www.commcarehq.org/a/nigeria-1/apps/view/d5b98f27d7ef43429a79f420f88c70af/form/adab5ea8b48f4f6abf5ed1c5594dc673/source/#form/group_indv/indiv_consented/ces/oth_side_effect) ^required^

[56. How did you hear about the recent MDA campaign? /data/group_indv/indiv_consented/ces/hear](https://www.commcarehq.org/a/nigeria-1/apps/view/d5b98f27d7ef43429a79f420f88c70af/form/adab5ea8b48f4f6abf5ed1c5594dc673/source/#form/group_indv/indiv_consented/ces/hear) ^required^

- Did not hear about MDA campaign 0
- Community Drug Distributor (CDD) 1
- Teacher 2
- Family member 3
- Community Leader 4
- Friend/neighbour 5
- Health Centre 6
- Public Address e.g. Loud speakers 7
- Radio 8
- Poster/flyer 9
- Television 10
- Place of worship 11
- Other (please specify) 99

[57. Other place you hear, please specify /data/group_indv/indiv_consented/ces/other_hear](https://www.commcarehq.org/a/nigeria-1/apps/view/d5b98f27d7ef43429a79f420f88c70af/form/adab5ea8b48f4f6abf5ed1c5594dc673/source/#form/group_indv/indiv_consented/ces/other_hear) ^required^

[53. Including the last MDA, how many times have you taken medication for Oncho? /data/group_indv/indiv_consented/ces/ivm_freq](https://www.commcarehq.org/a/nigeria-1/apps/view/d5b98f27d7ef43429a79f420f88c70af/form/adab5ea8b48f4f6abf5ed1c5594dc673/source/#form/group_indv/indiv_consented/ces/ivm_freq) ^required^

- Never 1
- One time 2
- 2-5 times 3
- 5-10 times 4
- >10 times 5
- Don't know/can not remember 999

[When was the last time you took ivermectin/Mectizan? /data/group_indv/indiv_consented/ces/last_comm_ivm](https://www.commcarehq.org/a/nigeria-1/apps/view/d5b98f27d7ef43429a79f420f88c70af/form/adab5ea8b48f4f6abf5ed1c5594dc673/source/#form/group_indv/indiv_consented/ces/last_comm_ivm) ^required^

[58. How happy are you with the drug distribution campaign that took place in your school/community /data/group_indv/indiv_consented/ces/satisfy](https://www.commcarehq.org/a/nigeria-1/apps/view/d5b98f27d7ef43429a79f420f88c70af/form/adab5ea8b48f4f6abf5ed1c5594dc673/source/#form/group_indv/indiv_consented/ces/satisfy) ^required^

- Not satisfied at all 0
- Not satisfied 1
- Satisfied 3
- Very satisfied 4

[59. Why were you happy with the drug distribution campaign that took place in your school/community? /data/group_indv/indiv_consented/ces/why_not_satisfy](https://www.commcarehq.org/a/nigeria-1/apps/view/d5b98f27d7ef43429a79f420f88c70af/form/adab5ea8b48f4f6abf5ed1c5594dc673/source/#form/group_indv/indiv_consented/ces/why_not_satisfy) ^required^

- Did not receive drug during the campaign 1
- Lack of information on the campaign 2
- Dislike for the time of the day of drug distribution 3
- Dislike for the month/period of drug distribution (farming or raining season) 4
- Occasional side effect 5
- Attitude of CDDs 6
- Other 7

[60. What can be done to improve the distribution? /data/group_indv/indiv_consented/ces/improvement_need](https://www.commcarehq.org/a/nigeria-1/apps/view/d5b98f27d7ef43429a79f420f88c70af/form/adab5ea8b48f4f6abf5ed1c5594dc673/source/#form/group_indv/indiv_consented/ces/improvement_need) ^required^

[61. This is the end of the survey, thank you for participating /data/note3](https://www.commcarehq.org/a/nigeria-1/apps/view/d5b98f27d7ef43429a79f420f88c70af/form/adab5ea8b48f4f6abf5ed1c5594dc673/source/#form/note3) ^required^
