# Supplementary material for: Reaching the last mile with ivermectin mass drug administration against onchocerciasis: The case of Kwanware-Ottou persistent transmission focus in the Wenchi health district of Ghana
Source: PLoS Negl Trop Dis. 2026 Feb 5;20(2):e0013958. doi: 10.1371/journal.pntd.0013958 (PMC12900439; doi:10.1371/journal.pntd.0013958)
Supplement: S2 Text — (DOCX) [file pntd.0013958.s002.docx]

Appendix 2: Qualitative assessment guides

1. **Qualitative Interview guide exploring challenges and solutions**

**FOCUS GROUP DISCUSSIONS (FGDS) WITH COMMUNITY MEMBERS FOR EVALAUTION OF ACTIONS IMPLEMENTED**

The following introductions could be used

***Thank you for agreeing to take part in this discussion. The objective of this discussion is to understand your experiences when the people came to give out the oncho medicine in your community. We are particularly interested in your understandings of the programme and how you feel about the way it is implemented in your community. As this is a group discussion we ask that you respect the opinions and confidentiality of others within the group.***

Ice Breaker

Begin by trying to make the group feel at ease. To do this, get them to introduce their experience of the most recent treatment round. You could do this by saying the following:

***I would like each of you to introduce yourself and tell me briefly about your experience during the most recent time that the people came to give out the oncho medicine.***

Once all the participants have shared their brief story with you, then tell the participants that we are going to explore the process of giving out oncho medicine in a little more detail.

**Topic Guidance**

The topics and questions below can be used to help guide your discussion. The questions have been divided into areas of Awareness, Availability, and Accessibility of the programme, as well as what could be improved.

AWARENESS OF THE PROGRAMME

Can you tell me what you know about the purpose of Ivermectin/Albendazole?

Did you know in advance of the distribution of medicines?

If yes, tell me what you know about it.

Who told you these things? E.g. Family, traditional healer, friends, health workers, CDD etc.

How were you told? E.g. using leaflet, verbally, CIC, radio/FM, TV, PAS, Mobile van announcements, poster etc. (What IEC materials or methods were used?)

Where were you told? E.g. at the health facility, church, mosque, market, funeral grounds, town meeting place, durbar, town chiefs house etc. (What structures were used to share awareness messages?)

How did this differ from previous times they have distributed the medicines in relation to awareness?

What did you like about this time in relation to awareness during MDA? What did you dislike about this time in relation to awareness during MDA?

AVAILABILITY OF THE PROGRAMME

Can you tell me what you think about the time of day/year that the distribution of Ivermectin/Albendazole took place?

What do you like about the timing for MDA? What do you dislike about the timing for MDA?

Can you tell me about any parts of the community or people within the community who were not available.

Can you tell me about any other time the distribution of Mectizan/Albendazole took place?

What did you think about that timing? What did you like/dislike about it?

Can you tell me about any parts of the community or people within the community who were not reached because distribution took place at this time of year?

During the most recent medicine distribution, was there enough medicines available to reach everyone who wanted them in the community?

If yes, how would you compare this to previous MDAs?

If no, who was not able to access the medicine because of this? What did people do to get community members to take the medicines?

Were there any delays in the medicines getting to the community? Why?/Why not?

**ACCESSIBILITY OF THE PROGRAMME**

**Can you tell us about the ways that the medicines were distributed during the most recent MDA?**

**What structures were used in the community during the distribution?**

- How were they used? (Probe for: giving out medicines, follow up etc).
- Is this the same or different from the previous MDAs?
- What period of day could you access the medicines?
- What did you like/dislike about the time for MDAs?
- What time would you like it to be done next MDA?
- Were there people in your community who couldn’t access the medicines because of the way it was distributed? Who were these people? Why could they not access the medicines?
- If people were absent from the community during the distribution, how were they followed up? Who followed them up?
- How much does it cost the community/individuals to be able to access the medicines?
- Do you pay to get the medicines?
- How much does it cost to get to where the medicines are being distributed?
- Do you have to take time from your routine activities to be able to access the medicines? If yes, how much time do you take?

**ACCEPTABILITY OF THE PROGRAMME**

**Can you tell us about how the people who distributed the medicines during this round of MDA were selected?**

**Who selected them? Where were they selected from?**

- Was this the same or different to previous years of MDA?
- Can certain people access certain parts of the community? Can men enter all the households? Can women enter all the households? Etc.
- What do you think about the people who were selected to distribute medicine?
- How knowledgeable/skilful are they?
- What activities do they or have they carried out to tell the community about the programme?
- Can you tell us any stories about people in your community who refuse to take the medicines during MDA?
- Which are the groups/individuals who refuse to take the medicine? Why do they refuse to take it? (Probe for: side effects, cost of the medicines, perception of need/traditional beliefs etc.)
- What could be done to encourage these people to take the medicines?
- What do you think are the advantages/disadvantages of taking the medicines?
- Can you describe the positive impacts you have seen because of people taking the medicines?
- Can you describe the negative impacts you have seen because of people taking the medicines?

**AREAS FOR IMPROVEMENT**

**What could be done better in the next MDA? Eg: Training, social mobilization, distribution etc.**

**What other ways do you think the distribution of medicines could have been done?**

- How were you involved in the last MDA?
- How could you or your community be better involved or prefer to be involved in the future MDAs?
- How have the MDAs or the programme made a difference to you, your family or your community?
- Closing the FGD

***Thank you very much for taking the time to answer my questions, do you have any questions for me?***

**Key Informant Interview guide with CDD and community stakeholders for the evaluation of actions implemented.**

**Introduction to Interview**

Below is some text that will help you to explain the purpose of the interview:

***Thank you for agreeing to take part in this research study. The objective of this discussion is to understand your experiences during MDA in your community. We are particularly interested in your understandings of the programme and how you feel about the way it is implemented in your community.***

Remember to familiarise yourself with the participant and make them feel comfortable in your presence.

**AWARENESS OF THE PROGRAMME**

**Can you tell me what you know about the purpose of Ivermectin?**

What did you know prior to the distribution?

What were you told?

Who told you these things? E.g. Family, traditional leader, friends, training sessions, health worker.

How were you told? E.g. using leaflet, verbally, poster, training materials etc. (What IEC materials or methods were used?)

Where were you told? E.g. at clinic, town chiefs house etc. (What structures were used to share awareness messages?)

How did this differ from previous times they have brought the oncho medicine?

- What did you like about it this time? What didn’t you like about it this time?

How easy is it for you to access and understand information about the medicines?

(Probe here for ability to read/understand/access awareness information, engaged with by CDD/health workers)

What would enable you to access information more easily?

What ways help you understand?

Where and how should the information be provided to you?

**AVAILABILITY OF THE PROGRAMME**

**Can you tell me what you think about the time of day/year that Mass administration of medicines for Mectizan took place?**

What do you like about the timing? What do you dislike about the timing?

How did the timing of the distribution help/hinder you in taking the medicines?

Can you tell me about any other time the distribution took place that is different from the recent?

What did you think about that timing? What did you like/dislike about it?

How did the timing of that distribution help/hinder you to take the medicines?

During the most recent Mass administration of medicines, were there enough medicines available to reach you?

If yes, how did this compare to other times it has been distributed?

If no, how did you access the medicines? What did people do to try to get you the medicines?

Were there any delays in the medicines getting to you? Why?/Why not?

Was there anyone in the community that the medicines did not reach? Why did it not reach them?

**ACCESSIBILITY OF THE PROGRAMME**

**Can you tell us about the ways that the medicines were distributed during Mass Administration of Medicines?**

**What structures were used in the community during the distribution?**

How were they used? (Probe for: giving out medicines, follow up etc.)

What times of day could you access the medicines? ***(CDD - What time of the day could you make the medicine available)***

What did you like about it? What did you dislike about it?

How much did it cost you to be able to access the medicines? ***(CDD – Cost of distributing the medicine)***

Did it cost you to get the medicine?

How much did it cost to get to where the medicines are being distributed?

What time did you have to take away from your routine activities to be able to access the medicines? ***(CDD - What time did you have to take away from your routine activities to make the medicines available to the community members?)***

What would make it easier for you to be able to access the medicines? ***(CDD – National, Regional, District, Subdistrict)***

Were there people in your community/household who couldn’t access the medicines because of the way it was distributed?

Who were these people? Why could they not access the medicines?

If people were absent from the community during the distribution, how were they followed up? Who followed them up?

If the participant was not able to access medicines during the last MDA, what was different about the way the medicine was distributed during the most recent distribution that allowed you to access the medicines?

How did you feel about being able to access the medicines this time?

**ACCEPTABILITY OF THE PROGRAMME**

**Can you tell us about how the CDDs were selected? – *(CDD – How were you selected?)***

Who selected them? Where were they selected from?

Is it the same way they have been selected before or different?

Can certain people from the community offer medicines more easily than others? Can men give you the medicines? Why/Why not? Can women give you the medicines? Why/Why not?

Who offered you the medicines?

What do you think about the people who were selected to give out the oncho medicine? ***(Stakeholder)***

How knowledgeable/skilful were they?

What activities did they carry out to tell you about the programme?

In future, who would you most like to receive medicines from? Why?

Why or why did you not take the medicines during this distribution?

(If participant didn’t take, probe for side effects, cost of medicines, perception of need/traditional beliefs, absent from the community etc.)

(If participant did take the medicine, probe for medicine benefits, feeling of wellness, who instructed them to take the medicines etc.)

Why did you take/or not take the medicine during the last round of distribution?

(If the participant didn’t take the medicines in the last round, but took them in this round): What was different about this medicine distribution that meant you accepted the medicines?

Can you tell us any stories about people in your community/household who refuse to take the oncho medicine?

Which groups or individuals refuse to take the medicine? Why do they refuse to take it? (Probe for: side effects, cost of the medicines, perception of need/traditional beliefs etc.)

What could be done to encourage these people to take the medicines?

What do you think are the benefits of taking the medicines?

**Can you describe the positive impacts you have seen because of taking the medicines?**

**AREAS FOR IMPROVEMENT**

**How could the distribution of the MDAs have been done differently? How would these changes help you to take the medicines? *(in terms of training, community sensitization, drug distribution etc.)***

**Key Informant Interview guide with Health Worker for the evaluation of actions implemented.**

**Introduction to Interview**

Below is some text that will help you to explain the purpose of the interview:

***Thank you for agreeing to take part in this research study. The objective of this discussion is to understand your experiences during MDA in your community. We are particularly interested in your understandings of the programme and how you feel about the way it is implemented in your community.***

Remember to familiarise yourself with the participant and make them feel comfortable in your presence.

**Awareness of the programme**

What did you know about the recent MDA?

Who told you? ***(Training)***

What were you told?

﻿How were you told? ***(format of the training)***

﻿﻿How easy was it for you to access and understand the information needed for the recent MDA?

﻿﻿Do you know about Adverse drug reactions of Ivermectin? ***(Probe for strategies put in place to attend to people with ADRs)***

**AVAILABILITY OF THE PROGRAMME**

Can you tell me what you think about the time of day/month/year that MDAs takes place?

What do you like about the timing? What do you dislike about the timing?

How did the timing of the distribution help/hinder the community members from taking the medicines?

Can you tell me about any other time the distribution took place that is different from the recent?

What did you think about that timing? What did you like/dislike about it?

How did the timing of that distribution help/hinder you to take the medicines?

During the most recent MDA, were there enough medicines available for the implementation?

If yes, how did this compare to previous MDAs?

If no, why were there not enough medicines? What did you do to try to get the medicines to the community members?

Were there any delays in the medicines getting to the community members? Why?/Why not?

Was there anyone in the community that the medicines did not reach? Why did it not reach them?

**Accessibility**

What would make it easier for you to be able to access the medicines for MDAs? ***(eg: from regional level/district level)***

**Acceptability**

Can you tell us about how the CDDs who gave out the medicines during this recent MDA were selected?

Who selected them?

***(Probing questions for refusal)***

﻿﻿Which were the groups/individuals who refused to take the medicine?

﻿﻿Why do they refuse?

What strategies are in place to attend to people with adverse reaction?

**Area of Improvement**

1. What could have been done differently during this recent MDA?

***(Probe in terms of training, community senstization, drug distribution)***

Appendix 2.2. Qualitative Interview guide assessing implementation of Action Plan

**Tell me about the general health issues in your community.**

*INTERVIEWER NOTES: If necessary, prompt with the following*

What are the main diseases/illness affecting the community?

How do the community resolve these health issues?

Where would they normally go when they have health problems?

What are the barriers to accessing this health care?

What health interventions do the community usually receive here- how and by who?

**Please tell me what you know about the disease (Onchocerciasis)**

*INTERVIEWER NOTES: If necessary, prompt with the following:*

Local names

How do one get it?

Tell me what happens to anyone that get the disease?

Tell me about the vector that transmit it

What do you think about this vector?

How and where do you know about the disease

Do you know anyone who has the disease, can you tell me about them?

*Do you think onchocerciasis (local name) it is a problem here?*

Who do you think gets the disease most? (Prompt: Men? Women? Children?)

*What is the belief of the community about how someone get onchocerciasis?*

*How does people in the community treat the onchocerciasis?*

*What is your feeling about the level of awareness of the onchocerciasis in the community?*

*Do you think the community has enough information about the disease and mectizan distribution?*

*What can be done to improve awareness?*

**Please tell me about Mectizan distribution in this community?**

How long has Mectizan been distributed in the community?

How often/frequent is Mectizan distributed in this community?

How does the community get involve?

What support do you get form the district Health office during MDA?

Do you take Mectizan? Why do you take it

Are there people that refuse to take Mectizan in this community?

What do you think is the reason they refuse to take mectizan

**Tell me about your role in the distribution of Mectizan (Ivermectin) in this community.**

*INTERVIEWER NOTES: If necessary, prompt with the following:*

For how long have you worked in this current role in this community/district?

How were you selected into the role?

What are your key responsibilities in this area of work?

What kinds of activities do you do? (prompt: census, delivery of treatment, report writing, information giving)

What support do you get from the community?

What challenges do you face in your role during MDA?

Were you trained for this role? If yes, how were you trained?

Which area and population do you cover?

Do you always meet your target? Prob the reason for the answer given

how would you rate the performance of MDA?

When was the last MDA in this community?

What challenges do you face in your role during MDA in this community?

What is the community perception and attitude towards the Mectizan distribution?

What are the cultural/religious issues in the community affecting MDA?

**Tell me what happens during the drug distribution period for (Onchocerciasis) in your community [DATE].**

*INTERVIEWER NOTES: If necessary, prompt with any of the following:*

How did it go?

What was the role of village leader, schoolteachers, church or mosque leaders, neighbors?

The approach used for distribution (House to house, gathering at a location e,g mosque, church, community square, palace

How far were you from the houses you needed to reach?

Where you able to reach your goals? (Why/Why not?)

**How cooperative is your community with the mass drug administration of** **(Onchocerciasis) in this area?**

INTERVIEWER NOTES: If necessary, prompt with any of the following:

Acceptance of volunteers or drug distributors

Timing of the MDA and the availability of the community (harvest/planting season, school holidays)

Community leader participation

Helping to facilitate the MDA (preparing water, food, helping volunteers/CDD)

**Sometimes people have side effects after they take the treatment for (Onchocerciasis). I’d like to ask you about any side effects that occurred here after the last community treatment on [DATE].**

INTERVIEWER NOTES: If necessary, prompt with any of the following:

Tell me about the side effects that you witnessed or heard about in your community.

Tell me about the reaction in the community to these side effects

Were there any more side effects than in previous years?

What advice do you have for the program to ensure that people understand why these side effects occur?

What advice do you have for the program to ensure that people feel reassured if there are any side effects?

**Please tell me what people did if they experienced side effects after taking the treatment for (Onchocerciasis)**

INTERVIEWER NOTES: If necessary, prompt with any of the following:

Go to the local health center

Solicited the aid of traditional healers

Prepared local home remedies

What do you think people in your community need to understand about side effects?

**In your opinion, what would you recommend so that your community is reassured about side effects that may occur following the treatment for (Onchocerciasis)**

INTERVIEWER NOTES: If necessary, prompt with any of the following:

Any specific communication about side effects?

Management of potential side effects by the health center?

**Thinking about your community, can you think of a group or groups of people who may be hard to reach (distribute the treatment for (Onchocerciasis)?**

INTERVIEWER NOTES: If necessary, prompt with any of the following:

Who are these people? (Prompt for Nomads)

Why are they hard to reach?

Are they hard to reach for other activities as well?

What advice do you have to reach this group/these groups in future distributions of drugs?

**In your community, can you tell me about any groups of individuals who are away/out of station for significant periods during the year?**

INTERVIEWER NOTES: If necessary, prompt with any of the following:

More men/women? Young/old?

Is it the same groups each year?

Are these people away during the same months each year (e.g., seasonal migration)?

How do you think this migration might affect their participation in the treatment for (Onchocerciasis)

**What do you think worked well during the last delivery of the treatment for (Onchocerciasis)?**

INTERVIEWER NOTES: If necessary, prompt with any of the following:

Training

Timing (day, period) of drug distribution

Side effects monitoring

Selection / training of CDDs

Social mobilization

Mopping up

Data quality and sharing

Availability of necessary materials and treatments

Social mobilization

Mopping up

**What suggestions do you have to improve the delivery of the next treatment (Onchocerciasis) in your community in the future?**

INTERVIEWER NOTES: If necessary, prompt with any of the following:

Timing during the day to deliver the treatments.

Side effects monitoring

Selection / training of CDDs

Time of the year (season) when MDA occurs.

Availability of necessary materials and treatments

**What suggestions do you have to promote health in your community in the future?**

INTERVIEWER NOTES: If necessary, prompt with any of the following:

Who do people listen to?

How are men and women reached differently with information?

What existing community structures (activities e,g society group, festival) can we leverage on for MDA programme

What is the role of community leaders in health education?

What advantage is the radio in health messaging? TV? Mobile phone?

**What do you think is the most important message for health in your community?**

INTERVIEWER NOTES: If necessary, prompt with any of the following:

Why do you say that?

Is it the same message for men and women?

**What suggestions do you have for the messages we might use to promote the next treatment for (Onchocerciasis) in your community in the future?**

INTERVIEWER NOTES: If necessary, prompt with any of the following:

What would motivate/encourage people to swallow the pills?

Who should deliver those messages?

In your opinion, do we need to make a new message?

**Tell me about the coordination of the MDA for (Onchocerciasis) in your community?**

INTERVIEWER NOTES: If necessary, prompt with any of the following:

How often?

Format of communication (text messages, phone, in person)

Who do you communicate most with?

How do you feel about the level of communication (sufficient/insufficient)?

**Tell me how you communicate when you have a challenge during the MDA for (Onchocerciasis) in your community?**

INTERVIEWER NOTES: If necessary, prompt with any of the following:

Who do you contact first?

Format of communication (text messages, phone, in person)

What happens when you share your challenge? Is it usually resolved (or not)?

**What suggestions do you have to improve coordination the next treatment for (Onchocerciasis) in your community in the future?**

INTERVIEWER NOTES: If necessary, prompt with any of the following:

Timing issues

Format of communication (text messages, phone, in person)

Information-related to the MDA

**Tell me about the materials and treatments (drugs) you need for the treatment for (Onchocerciasis) in your community?**

INTERVIEWER NOTES: If necessary, prompt with any of the following:

Timing issues—receipt of IEC materials and drugs ahead of MDA?

Stationary items (pens, paper, etc.) for reporting

Sufficient numbers of tablets/treatments (how was this determined?)

Sufficient numbers of IEC materials—how do they decide who gets them?
