# Supplementary material for: Reaching the last mile with ivermectin mass drug administration against onchocerciasis: The case of Kwanware-Ottou persistent transmission focus in the Wenchi health district of Ghana
Source: PLoS Negl Trop Dis. 2026 Feb 5;20(2):e0013958. doi: 10.1371/journal.pntd.0013958 (PMC12900439; doi:10.1371/journal.pntd.0013958)
Supplement: S3 Text — (DOCX) [file pntd.0013958.s003.docx]

*Appendix 3. Findings, accompanying actions, successes, and challenges of implementation*

| **Findings and issues identified** | **Actions implemented** | **Successes** | **Challenges/ action to improve** |
| --- | --- | --- | --- |
| The population recorded in community registers was much higher than the census population | - Re-registration of all households - NTDP provided new registers and conducted electronic registration | - Actual population counted. - Strengthened collaboration among community, CDD, and frontline health workers | - It was challenging reaching everyone as some were absent - CDD had to undertake 2-3 follow-up visits |
| Poor ivermectin reach/uptake in remote communities  Low reach/uptake of ivermectin among miners and cattle herders (Fulanis)  Absences due to daily and seasonal, mobility or migration: daily (to work, farm); travel outside community, visitors, recent arrivals  Twelve settlements not distinguished in previous MDA records were identified. | - Community health workers and CDDs conducted outreach visits to communities - Health workers and CDDs met with key stakeholders in small-scale mining and Fulanis settlements to raise awareness and secure their adherence to MDA - District and sub-District staff conducted 3 supervisory visits to these communities - NTDP staff conducted 2 supervisory visits during the MDA - Communities informed in advance of the distribution dates | - Improved community participation leading to high ivermectin treatment uptake - A Fulani CDD was recruited and paired with an experienced CDD - NTDP District Management Tool was updated to include 7 additional communities - The Action Plan was integrated into NTDP Tools to guide reflection before and after MDA | - Some household members were not present during the distribution period, so CDDs kept ivermectin or obtained from the district and offered to returning community members - Health workers and CDDs leveraged on community resting day for raising awareness and offering treatment - District Assembly did not grant audience before MDA. So, correspondences were sent twice. |

*Appendix 3 continues. Findings, accompanying actions, successes, and challenges of implementation*

| **Findings** | **Actions implemented** | **Successes** | **Challenges/ action to improve** |
| --- | --- | --- | --- |
| Low awareness and misconceptions  Some community members are never treated (NT).  Some community members refuse to take ivermectin for fear of side effects | - Conducted awareness talk in community ‘durbar’ (meetings) and during child welfare clinics in the communities - Used ‘gong-gong’ & and megaphones to raise awareness in the community - Held one-to-one awareness talks with individuals who have never been treated. - Reinforced community education on ivermectin side effects and provided assurance of freely available support | - Strengthened community collaboration, - Improved community participation in MDA - Increased awareness of transmission, treatment and management of side effects - No treatment refusal reported | - Not all community were visited due to inadequate resources. - Inadequate posters for community awareness |
| Lack of CDD in some communities and inadequate training  Low quality of MDA implementation (not using dosing pole, no directly observed treatment, incorrect recording of treatment) | - Recruited 14 new CDDs - All CDDs trained on sensitisation, registration, administration and recording of treatment. - Supplied measuring/dosing poles. | - NTDP used Action Plan as a guide to reinforce regional and district planning and training |  |
