# Supplementary material for: Reaching the last mile with ivermectin mass drug administration against onchocerciasis: The case of Kwanware-Ottou persistent transmission focus in the Wenchi health district of Ghana
Source: PLoS Negl Trop Dis. 2026 Feb 5;20(2):e0013958. doi: 10.1371/journal.pntd.0013958 (PMC12900439; doi:10.1371/journal.pntd.0013958)
Supplement: Attachment — Submitted filename: Response_to_Reviewers.docx [file pntd.0013958.s007.docx]

Dear Editors,

We sincerely thank the reviewers for their thoughtful and constructive feedback on our manuscript. We have carefully considered each comment and made substantial revisions to address the concerns raised. Below are detailed point-by-point responses.

Sincerely,
Rogers Nditanchou (on behalf of all co-authors)

**Reviewers' Comments:**

Reviewer's Responses to Questions

**Key Review Criteria Required for Acceptance?**

As you describe the new analyses required for acceptance, please consider the following:

**Methods**

-Are the objectives of the study clearly articulated with a clear testable hypothesis stated?

-Is the study design appropriate to address the stated objectives?

-Is the population clearly described and appropriate for the hypothesis being tested?

-Is the sample size sufficient to ensure adequate power to address the hypothesis being tested?

-Were correct statistical analysis used to support conclusions?

-Are there concerns about ethical or regulatory requirements being met?

Reviewer #1: Objectives of the study are clearly articulated; population is clearly described; ethical requirements were met.

One problem is that the assessment of efficacy was entirely qualitative. The standard in the field is to do comparative surveys, where people are asked to respond to the same series of questions so that comparisons can be made across time points in a statistically rigorous manner, and allowing for assessment of sample size relative to population size etc. Focus groups are not a good method for assessing changes in attitudes, because people's responses in groups are impacted by what is expressed by others around them, and because one speaker could monopolize the response time and their opinions become overly reflected in the resulting transcripts.

The authors clearly state that their assessment is qualitative, and this study is now over so I'm not necessarily suggesting that surveys be done now. However, one of the main goals of the study is to provide an approach for developing action plans that could be applied elsewhere, and assessment of efficacy should be an important component of any action plan. Methods for assessment need to be rigorous and applied both before and after the implementation of the Action Plan. It looks to me like the Census included some parts of a knowledge/attitude/practice survey, in that they asked why people didn't take the drug and whether they knew about MDA. Could the CDDs do phone-based surveys as part of their duties, for example, or is this placing too much of a burden on training?

We appreciate the reviewer’s insightful critique regarding the limitations of qualitative approaches in assessing efficacy. In the revised manuscript, we have clarified that while focus group discussions (FGDs) offer valuable contextual insights, they are inherently susceptible to bias due to group dynamics and the dominance of individual voices. As such, their reliability in measuring attitudinal change is limited (Lines 550–576).

Nevertheless, facilitators actively managed group dynamics to prevent any individual from dominating discussions. Potentially dominant participants were invited to speak last, allowing others to contribute first. Based on field experience, individual interviews in rural communities were less effective due to participant shyness or fear. Group settings encouraged freer expression, often influenced by peer interaction. Additionally, community attitudes toward interventions were frequently shaped by the views of local leaders rather than personal opinions. See lines 187-193.

However, we have also presented quantitative findings that support and align with the qualitative results, thereby strengthening the overall interpretation of impact. See lines 440-453; Table 3, line 454-459; lines 524-527

A sample size calculation was not required, as the census included the entire population, ensuring high precision (see Lines 166-167). We now explicitly highlight that the census captured key knowledge, attitudes, and practices (KAP)-related metrics, which partially address this methodological need (see Lines 264–302).

Why was 10km chosen as a radius? Is there evidence that this range is useful or relevant to oncho transmission (i.e., a transmission zone) or is it arbitrary? Looking at Figure 3, there are communities that appear to be on the border/overlap with the 10km radius.

We have clarified in the introduction of the manuscript that the 10 km radius was informed by findings from our previously published study, which delimited the transmission focus based on entomological and epidemiological findings. Lines 99-103.

The Intervention was implemented at the sub-district level for operational reasons and include all communities within or near the boundary of the 10 km radius to ensure comprehensive coverage of the focus and alignment with health system organisation. We have updated the manuscript to clarified this. See lines 115-117; 142, 145; 598-599.

Having the same person run the interviews and do the transcriptions could introduce errors as there is no independent evaluation. I'm not a social scientist but perhaps check best practices in the field for this.

Thank you for this thoughtful observation. We appreciate the concern of the reviewer; however, It is actually common practice in qualitative research for the interviewer to undertake the transcription of the audio files into transcripts for analysis before audio-recording became available, ethnographers would actually go out, conduct interviews, take notes during the interviews and later in the evening develop those notes into detailed transcripts for analysis. Nevertheless, all transcripts were independently reviewed and verified by a second researcher to ensure accuracy and reduce interpretive bias. We have updated the manuscript. See lines 230-233; 550-556.

Reviewer #2: The Methods are adequately described.

*Thank you.*

**Results**

-Does the analysis presented match the analysis plan?

-Are the results clearly and completely presented?

-Are the figures (Tables, Images) of sufficient quality for clarity?

Reviewer #1: Need to indicate the time scale of each step--when was MDA? when did the first FGD occur? When did meeting with other stakeholders occur? When was the plan developed and who was in the room when that happened? When was the plan implemented? When did MDA occur? Who assessed reported coverage? When were the second FGD/KIIs held and how was it decided who to interview?

Thank you. We have now added activity time scale. See Table 1, lines 135

The selection of participants for stakeholder meetings and subsequent interviews was done in collaboration with the program team, based on individuals’ involvement in MDA and perceived influence within their communities. Furthermore, findings from the Coverage Evaluation Survey (CES) and infection status informed the purposive selection of interviewees, ensuring inclusion of those with low participation and high infection rates to better understand barriers and identify ways to enhance their engagement. Lines 176-182.

Reported coverage was assessed by community members, subdistrict, district, and national staff. See lines 210-212. Additionally, it was further reviewed during the action plan refinement meeting. See line 461-465.

Could the quantitative results of why people didn't take the drug be reported in this section?
Thank you for this helpful suggestion. We have now added these in the revised manuscript. See lines 264-282 and Figure 4 (new).

Refinements: who were the stakeholders involved in this workshop? How many people attended relative to the earlier workshop?

Thank you. There were 20 participants in the refinement workshop and 30 in the development workshop. This difference was due to financial constraints rather than lack of interest or refusal. Stakeholders who attended both the action plan development and refinement workshops are listed in the revised manuscripts. See lines 198-201; 213-217.

How determine how many additional CDDs are needed and where they should be located? Is there an existing protocol for this that is implemented in Ghana?

To our knowledge, there is no nationally standardized protocol for determining the number or distribution of Community Drug Distributors (CDDs) in onchocerciasis-endemic settings in Ghana. In this study, recruitment was guided by practical feedback from field implementation, where communities reported either the absence of CDDs or excessive workload on existing personnel. Decisions regarding additional recruitment and placement were made collaboratively with district health teams based on perceived feasibility and operational needs. While some programs informally apply ratios such as one CDD per 100 households, such benchmarks are context-dependent and not universally adopted. We acknowledge this limitation and recognize the value of establishing structured guidelines to support future programmatic planning and equitable distribution of CDDs. See lines 556-567.

Appendix 3 is not the Action Plan, but a summary table that indicates the actions taken and by whom. But the Action Plan itself was meant to indicate who was responsible for what action and how implementation success would be assessed? It would be good to see that document.

Thank you for the clarification. The Action Plan has now been included as Appendix 4. Line 412.

Reviewer #2: The authors describe this as a mixed methods study utilising a combination of qualitative and quantitative analyses. My reading of the Results section of the manuscript is that there are almost no quantitative data at all.

Thank you for this valuable observation. The census, including the CES, provides quantitative data (see lines 257–290). Additionally, we have now included post-intervention reported coverage to further strengthen the evidence of the intervention's impact (see lines 454–459).
For example,
• in the section "A review of community treatment registers" there are qualitative statements such as "the number of people included in registers was consistently higher than the numbers counted in the census” but there is no quantitative estimate of how much higher, nor of what “consistently” means. Consistently between communities? Consistently between occupations? Genders? All of which are shown later to be associated with coverage.

Thank you for your insightful comments. Our original intention was to review and update existing community treatment registers; however, this proved unfeasible due to significant data quality issues. See lines 168–174.

We have revised the manuscript to improve clarity, incorporated available quantitative estimates, and clarified the implications of overestimating the eligible population on therapeutic coverage. These revisions aim to strengthen the mixed-methods approach. See lines 257-290; 454–459; Table 3.

It is also worth noting that overestimating the number of people eligible for treatment would likely result in an underestimate of true coverage. Given that therapeutic coverage is a key parameter, the scale of the effect that the “consistent” overestimate of the size of the eligible population may hve on coverage reporting should be explored.

Thank you for your comment. We have now addressed this point in the manuscript (see . However, we acknowledge that only a comprehensive review of the old registers can quantitatively reveal the full extent of the issue. See lines 485-506).

• Table 1 reports that 3,338 people were censused in 19 communities. Does “people” refer here to all persons (irrespective of MDA eligibility status) or does it refer only to those people deemed to be eligible for treatment?

Thank you for this observation. The figure of 3,338 people censused in 19 communities refers to all individuals recorded during the household census, regardless of their eligibility for Mass Drug Administration (MDA). This total includes both eligible and ineligible individuals (e.g., children under the eligible age, pregnant women, or those very sick). See lines 257-258

Table 1, now updated as Table 3, has been revised accordingly. See lines 454-457.

And how does this number compare with the inflated estimates described qualitatively in the section above?

Given that the Action Plan was implemented at the sub-district level—with clearly defined geographic boundaries—we conducted population comparisons at this level Direct community-level comparisons were not feasible, as programme data pooled some communities, including those outside the focal area. Therefore, sub-district analysis provided the most reliable basis for comparison. See lines 441–453.

We compared pre-intervention reported coverage and survey coverage to illustrate coverage over reporting. See lines 241-253

Survey and post intervention sub-district coverages were compared to show improvement in coverage. See Table 3 and lines 454–459

More importantly, the therapeutic coverage for each community in March 2024 is given but there are no data for earlier MDA rounds, so there is no way of knowing whether, and in which direction, there is a change in MDA coverage.

Thank you for your observation. Due to data quality issues in the old registers, we instead compare survey coverage with post-intervention reported coverage. See Table 3 lines 254-259 and lines 420–430

To add to this uncertainty concerning how these data are to be interpreted, the text above the table refers to MDA in January 2024 whereas the Table caption refers to March 2024 and cites the same overall coverage (70.8%). So, do the text and the table report the same MDA round? If so, then when did this round actually take place?

Thank you for your observations. We have now made the necessary corrections. See lines 257-260 and refer to Table 1 (lines 135) for the timeline and Table 3 (454-459) for survey and post-intervention coverage data.
• Still on the question of the January/March 2024 coverage data, how does the reported overall coverage of 70.8% compare with the previous round(s)? These data are reported as people who “indicated they swallowed ivermectin in the recent round”. These data are therefore “survey data” i.e. data on coverage collected by asking people directly whether they were treated or not after the MDA round. This is in contrast to the earlier statements referring to treatment records from register data. It is well known that reported MDA coverage from community treatment registers almost always overestimates survey coverage which is measured by questioning people directly.

Thank you. We have now included data from the preceding MDA, which illustrate the extent of overestimation prior to the intervention. Lines 254-259.

• Taken together, my comments above point to at least three potential problems with the reporting of coverage in this section. First, the unknown extent of overestimation of the eligible population in the old inaccurate treatment registers should lead to an underestimate of coverage calculated from these registers.

While we appreciate your concern regarding the overestimation of the eligible population, we respectfully disagree with the suggestion that this would necessarily result in an underestimation of therapeutic coverage. Our findings indicate that individuals who were no longer present in the community—due to migration or death—were still recorded as treated in the registers. More concerningly, it appears that some individuals who were previously recorded but were not reached by the CDDs may also have been incorrectly marked as treated. Furthermore, some were present and not registered and so are not captured in any register-based coverage (see line 267-269). As a result, the numerator in the coverage calculation is inflated with inaccurate data, making it more likely that the reported coverage figures overestimate the actual treatment coverage. See lines 485-506.

We have now estimated the level of overestimation of population. See lines 254-259; 455-457; 493-503

Second, recording dead or absent people (in the old registers) as being treated would have the opposite outcome i.e. inflating reported coverage. So, which of these opposing features of the historical coverage estimates is important?

As discussed above, it is not only that individuals are recorded as present, but they are also marked as treated. This can lead to an overestimation of coverage, especially when individuals listed in the register but absent during the MDA are falsely recorded as having received treatment. Additionally, individuals who were present but not registered will have no impact on register-based coverage estimates, although they represent an epidemiologically important group. Such discrepancies inflate coverage figures and mask actual gaps in program reach. See line 497-503.

What, for example is the effect on the historical reported coverage of simply removing the dead and absent from the registers?

Programmes are designed to report on register-based coverage. While data cleaning can help remove individuals who are deceased or absent, this process is daunting because such statuses are rarely indicated in the register. These individuals were mostly falsely recorded as treated. If these cases were removed, coverage figures would drop to a more realistic level. See lines 497–503.

And third, the “current” coverage data reported in the table cannot be compared directly with the historic coverage estimates because they are “survey coverage” data derived from direct questioning of eligible people whereas the historic data are “reported coverage” from treatment records. These two methods of estimating coverage almost invariably generate different coverage estimates. My view is that we can conclude nothing concerning the impact of measures taken to improve coverage from the coverage data that are in the manuscript.

First, the historical coverage from the January MDA was compared to the survey results for validation, which is the standard practice (see Table 3, lines 258–281). Secondly, the survey coverage was then compared with the reinforced MDA conducted in July. See lines 450-451 and Table 3, lines 454-459

The July round involved a new registration process, was directly observed (DOT), and benefited from intensified field supervision. Monitoring was carried out by community members, sub-district, district, and national teams, making it comparable to the survey coverage. See lines 520-530.

However, we do acknowledge that conducting another survey would have provided a more robust comparison. See lines 555-556.

The next section of the manuscript deals with qualitative assessment of challenges and solutions. The authors state in the Methods that they used the Nvivo package to analyse the qualitative responses collected from focus groups and discussions. I am not a social scientist and have not used the Nvivo package, but the description of the package states clearly that “offers tools for creating visual representations of data, such as word clouds, concept maps, and charts, facilitating data interpretation and presentation”. This section of the Results makes no reference at all the Nvivo analysis, nor does it contain any of the outputs and data display options that can be generated by the software as aids to more objective, semi-quantitative summaries of the discussions of focus groups etc. What are included in the manuscript are a series of verbatim quotes from focus group discussion participants. How representative are these isolated quotes of the discussion in general? Why did the authors include these specifically? What criteria were used to select these quotes? Are all the transcripts of all the discussions (i.e. the “raw data”) available? If so, where? If not, why not?

Thank you for your thoughtful feedback.

1. Use of NVivo Software:
   NVivo (version 14) was used to organize and manage coding of qualitative data. While it offers visual tools it is primarily interpretive, and it is not a standard reporting practice. We opted not to include them, as a quantitative assessment has now been added (see lines 440-459).
2. Verbatim Quotes:
   Quotes were selected to illustrate key themes aligned with study objectives and quantitative findings. This is now clarified in the manuscript (lines 236–238).
3. **Transcripts and Raw Data:**
   Transcripts were used to extract quotes and reflect identified themes. It is not standard practice to share full transcripts due to confidentiality concerns and so they are not publicly available. However, they can be shared upon reasonable request.

Other questions that come to mind with respect to this section are:
• the authors state that cattle herders rarely take MDA. However, Table 2 includes “cattle rearing” as an occupation that has a 70.3% MDA rate (compared with 70.8% for the whole population). The basis for the statement that cattle herders rarely participate appears to be the result of assuming that all cattle herders are members of the Fulani ethnic group. Where is the evidence for this?

Thank you for this important observation. Our original statement was based on qualitative observations and field reports indicating that nomadic cattle herders—particularly those from the Fulani ethnic group—are often absent during MDA campaigns due to their mobility and seasonal migration patterns. However, we recognize that not all individuals engaged in cattle rearing are nomadic or Fulani, and that the occupation category in Table 2 may include settled cattle rearers who are more likely to be present and participate in MDA. We also acknowledge that the assumption linking all cattle herders to the Fulani ethnic group was not explicitly supported by data in the manuscript. We have revised the text to clarify that the statement refers specifically to nomadic cattle herders. See lines 339-340.

• lack of community drug distributers: there are recommendations for the ratio of CDD’s per community and/or head of population. What are the numbers in these communities? How far below the recommended number of CDD’s for this size of target population. Likewise, there are guidelines for training frequency and content. What are the training records for CDD’s in these communities? What does lack of training for some CDD’s mean? What proportion of CDD’s do not receive training i.e. what does some mean? Is it a sufficiently large proportion that it is likely to impact MDA? The qualitative statements made here should be able to be supported by quantitative data on CDD numbers, rates of turnover and training that can be related to MDA differences between communities. The qualitative descriptions and anecdotal quotations in the manuscript are of little value in identifying which components of MDA delivery are most likely to contribute to poor outcomes.

We recognize the critical role of CDDs in successful MDA delivery and agree that more detail is needed. While a full audit of CDD deployment and training was not conducted, these operational decisions—including recruitment and training—were made by the implementing program. Action plans identified areas needing additional recruitment and training based on volunteer availability, population coverage, geographic feasibility, and resource needs. We have acknowledged this limitation see lines 556–567.

Lastly a couple of questions about Table 2.
First, what are the numbers in parentheses? I assume it is the number of people in each category. However, if this is the case and the category “All” 80.3% (1467) means there were 1467 people 15 years and older, why is the category “Permanent resident - did not travel 1603? What is the denominator for the percentage calculations? Again, in the Residency category, “Permanent resident – did not travel is 83.8% (1603) but “Permanent Resident – travelled is 55.9% (76). I cannot make sense of these numbers. Could the authors please explain how the percentages are calculated and what the number in parenthesis after each percentage means?
Second, the footnote to the table states that only people over 15 years old were included to “ensure a fair comparison across of variables”. What does this mean?

Thank you for your observations.

1. Numbers in Parentheses: These represent the number of individuals in each subgroup who took ivermectin. The percentage is calculated using the total number in that subgroup. The figure 1603 was an error and has now been corrected. The table has been updated with the correct data.
2. Age Restriction: Only participants aged 15 years or older were included to ensure they had been age-eligible for ivermectin treatment for at least 10 years, starting from the age of 5. This criterion was applied to ensure that age did not exclude individuals from any category of analysis variables.

The table has been updated accordingly. These clarifications are now included in the footnote of Table 3. See lines 295-298.

I have similar objections to the entirely anecdotal, qualitative way in which the implementation, evaluation and refinement of the action plan are described. The most obvious measure of the success of a plan for improvement would be an increase in therapeutic coverage following implementation of the plan. No such data are included. Without these data it is impossible to draw any conclusions as to whether the barriers to MDA implementation were identified correctly nor whether the remedial actions were effective (nor, indeed, whether this qualitative approach to improving MDA is itself effective).

Thank you for your comment. We have addressed this concern by including quantitative data to demonstrate the effectiveness of the intervention. With these additions, we found that the qualitative findings were coherent with the identified barriers, proposed solutions, and observed improvements in therapeutic coverage, thereby reinforcing the validity of the approach and the conclusions drawn. See lines 440-459; 520-530.

**Conclusions**

-Are the conclusions supported by the data presented?

-Are the limitations of analysis clearly described?

-Do the authors discuss how these data can be helpful to advance our understanding of the topic under study?

-Is public health relevance addressed?

Reviewer #1: This section was not very well organized, with sentences not really following from the sentence that came before it. Paragraph 2, for example, goes back and forth between topics; paragraph 3 was confusing, moving between treatment coverage, assessments, and uptake without making clear logical links. The discussion about the involvement of the NTD programme seemed like it should have come earlier?

I would encourage the authors to consider the discussion less as a reporting of the results from this region of Ghana, and more as making a targeted recommendation for how an Action Plan can be developed in conjunction with community and NTD programme people based on the experiences of this pilot. For example, start with how the authors would recommend that people in other countries determine the size of the area that should be included in developing an Action Plan? How should relevant stakeholders be determined? What are the challenges in identifying who should be involved? How should appropriate assessments be developed so that quantitative data could be collected before and after implementation? How should workshops be designed to encourage stakeholder participation? Highlight what this pilot did well and where improvements could be made for future adoptees of the approach; while as the authors note this isn't a cost study, it might be helpful to indicate what would be ideal vs what would be necessary (or at least strongly recommended).

Thank you for the insightful feedback. Our discussion have been revised to align. See discussion section lines 474-601.

Reviewer #2: The first paragraph of the Discussion summarises the methodology into 6 steps. The first of these (GIS + community consultation to find all the settlements in the study area) is obvious, is reported well and is appropriately discussed. The next 2 steps are essentially quantitative in nature (counting people by census and by consulting treatment registers) but no quantitative data or analyses are presented. Without quantitative reporting, the interpretation and discussion of these steps is not credible. There is no evidence presented to support the conclusions that are drawn.

Thank you for highlighting this important issue. We have revised the discussion to address the lack of quantitative. See lines 485-530.

I was completely lost when I reached the point in the Discussion that reads “Better MDA quality and performance was achieved as a result of action plan implemented. A 25.2% (1,023 treated individuals) increase in the number of persons treated was achieved during the MDAs in July compared to January 2024”. I simply could not find these critical data that apparently report the coverage in July 2024 (noting again that the coverage data in the text are referred to as January 2024 and also as March 2024, but certainly not July). Where are they? None of the Discussion that follows concerning the success of this approach to improving MDA coverage has any meaning unless there is rigorous quantitative data to support the claimed increase in MDA coverage, and data (preferably quantitative structured survey data rather than anecdotal focal group discussion reports) that linked the observed increase in coverage to specific actions.

Thank you for this important observation. We have revised the text to ensure consistency in the reported dates and have clearly presented the coverage figures for July 2024. Please refer to Table 1 (lines 135) and Table 3 (lines 454–479) for the quantitative data. Furthermore, Table 3, together with the updated Action Plan (Appendix 4), illustrates how specific interventions contributed to the observed increase in coverage. While the quality of implementation coverage, as justified in the manuscript, was adequate for comparison with survey coverage, we recognize that a post-implementation survey would have provided stronger validation. We have noted this as a recommendation for future work. See lines 555-556.

**Editorial and Data Presentation Modifications?**

Use this section for editorial suggestions as well as relatively minor modifications of existing data that would enhance clarity. If the only modifications needed are minor and/or editorial, you may wish to recommend “Minor Revision” or “Accept”.

Reviewer #1: Minor comments:
Background:
p4
remove parenthesis "onchocerciasis transmission by the year 2030)" Done; line 70.
indicate when OCP ended. Done; lines 77-78.
p5
6.7% and 5.9%. We have maintained ‰ (for per thousand). This can be checked in the reference provided. Line 91.
"2021 investigation revealed that the NTD" add "programme's". Done; line 94.
"inability to reach some at-risk populations". Done; line 94-95.
"including (illegal) miners, were". Done; line 94.
remove also and fix typos: "over the years were contributing to persistent on-going".

The sentence has been restructured, see line 96.
"We determined in our 2021..." The sentence has been restructured; line 99.

Methods:
p7 Figure legend: "10 km radius delimits". This has been modified. See Figure 1.
"treatment coverage evaluation surveys". Done; line 130.
p10 "a month following preceding MDA" --wording is confusing. Is this a typo? A month after MDA but before implementation of the action plan? Both before and after MDA?. Done; line 159.
p11 "During the workshop, an action plan" Done; line 196.
"Step 7: Implementation and evaluation of action plan" (not plans). Done; line 205; now step 6.
p12 "Commcare platform and cleaned and analysed using STATA". Done; line 223-224.
Remove definition of percentage "Percentages were calculated as...". Done; line 226.
(odds ratio, OR) not odd. Done; line 223.
Fix typo: "To ensure quality of transcription, research assistants that conducted"...Done; line 231-233.

Results:
p12
add mdash "... Akura, and Mambeele)-were identified and included." Done; line 246.
Already indicated settlements are remote; "These hamlets have poor accessibility..." Done; line 246-247.
The text reads that these settlements are "often assimilated into bigger communities for CDTI". Because this statement refers to actual settlements, rather than small remote settlements in general, there should be data on how many of the ten settlements and in how many rounds of CDTI they were assimilated--i.e., quantitative rather than qualitative. Thank you. We have indicated that all the ten were assimilated into larger settlement in preceding CDTI round. Lines 246-247.
p 13
"The outcome of the first few days" -- first few days of what? Of field verification, line 249. I'm confused
Regarding the "shacks", I'm confused by this term--do people not live in shacks? I feel like this term is being used as if it is referring to a specific type of dwelling that is not a temporary shelter? We have now removed “shacks” and updated. Line 251-252.
p14
"those who had died or moved away were still". Done; lines 170-171
Capitalize Table 1. Now Table 3. Done; lines 454
"uptake 0.26" is this a % or proportion? Looks like a proportion--I would recommend being consistent between reporting % or proportion in table 1 and text please. This is odds ratio. We have clarified this in Table 2, lines 293-298 and in the text, lines 284-286; p15
capitalize Table 2. Done; Table 2 -line 293
Table 2--Coverage column: indicate what number is in (). Is this the number of people that were covered or the total number of people in the category? The percentage represent the proportion of individuals within each subgroup who took ivermectin along with the actual number in parenthesis. We have updated. See Table 2 -lines 295-298

Does Age have a reference? Why is there no OR for each and only for age 35-44?

Thank you for your question. As indicated in Table 2 line 293, age was treated as a continuous variable in the logistic regression model. This means the model estimates the effect of each additional year of age on treatment uptake, rather than comparing predefined age categories. As a result, there is a single odds ratio representing the change in odds per year increase in age, rather than separate ORs for each age group. See table legend 295-298.

Residency -- remove carriage return and uncapitalize "travel outside the district". Done See Table 2 -lines 293

Fix typos: *15 years cut-off age ensured a fair comparison across variables. Corrected; lines 295-298.
p17
"assessment revealed context-specific challenges". Corrected; line 300.
"interviewees which may have an influence". removed; lines 303-308
p18
"community members included the belief that the disease is gotten from a contaminated" Corrected; lines 306-308.
Has "IDI" been defined? Corrected to KII (key informant interviews). See Table 1 135 for definition and line 319.
The suggestion that cattle herders rarely participate in MDAs because of seasonal migration is not supported by the provided statement. Is this speculation by the authors or is it something that was in the interviews? We have corrected this. Line 339-340.
p19
"Community members reported that the absence". Corrected; line 346-347.
Indent last quote on page for consistency. Corrected, line 372.
p20
Fix quote and spelling in second quote. Corrected; lines 384-389.
Remove comma "participation in MDA in the communities is the fear" Corrected; lines 380
"after taking the medicine and decided they would not take" Corrected; lines 382-383.
p21 - community members wanted more CDDs; how many is more? indicate how this was changed in the Action Plan if it was.

Thank you for your comment. While community members expressed a desire for more CDDs, the exact number was determined by the programme, taking into account factors such as population size, settlement distribution, and accessibility. The specific number of CDDs allocated to each area is detailed in the Action Plan, which reflects these considerations and adjustments made in response to community input. Lines 406-410; 556-567; Refined Action Plan -Appendix 4.

"Participants suggested that the community should be informed". Corrected; lines 321-322.
Add quotes around last quote on page. Is this community Kwadum Nsuta, Kwadum Joe, or a third community? Thank you. Kwadum Joe boy and the quotes have been corrected. Lines 323-325.
p22
Is this a boy? can CDDs be under 18? or is this to indicate male? It is name of a community; Lines 325
"Community members therefore recommended" Corrected; lines 377.

..." This would ensure that the true"..."persons who do not take or refuse to swallow the drug would be easily identified" Corrected; lines 378.
"mobilized the community effectively and changed the community's". This has been updated. See lines 396
The last paragraph of this page needs to be better organized. Start with what people were involved, then what the action plan included, then what was in the action plan. Thank you. We have restructured the paragraph; lines 400-413
"hard-to-reach groups, including" Modified line 403.

consistent spelling for sensitization/sensitisation. I also don't know what the authors mean by this word no matter the spelling?

Thank you for your comment. We have replaced the term "sensitisation" with "awareness" throughout the manuscript for clarity and consistency. This change better reflects the intended meaning, which refers to activities aimed at informing and educating community members about the MDA process, its purpose, and benefits. See lines 303-306.

See detailed Action Plan in Appendix 3. We have changed this to “Further details are provided in Appendix 3”; line 471-472.
p 23
Implementation and evaluation of action plan -- this section is very thin, only one sentence! Perhaps remove subheading of qualitative assessment? Thank you. We have reorganised this section. See line 390; 403
Census using electronic registration should be noted in the section above
Thank you. We have reorganised this section. See lines 400, 412,

Is there a bias in who comes to the FGD?

Thank you. There was a sampling bias toward selecting people with the most relevant information. So, participants were purposefully selected based on their involvement in MDA implementation, or because they were reported as infected or missed during the MDA. This approach enabled the collection of targeted information relevant to programme improvement. See lines 176-182.

When were the FGDs run relative to the implementation? What is the time scale of any of this?

We have provided activity timeline in Table 1. See lines 135.

capitalize Kwandum. Thank you. This now capitalised. Lines 423, 463.

p24
"Participants also highlighted areas for" Thank you. it is now corrected. See line -434.
what is a "mop-up"? this is a fairly casual term where I come from. Perhaps "follow-up"? Thank you. it is now corrected. See line -434
check spelling of "wifse". Thank you. This now corrected. Line 438.
check capitalization of Kwadum. Thank you. This now corrected. Lines 403, 463.
Add consistency with whether Action Plan is capitalized or not. Thank you. We have corrected here and abbreviated as AP. see Table 1, line 135; lines 194 and throughout.

p25.
"no study has combined” We have removed this. – 474-484

"identification of 12 settlements that were not identified in” removed line 255
"masked the need for CDDs as well as straining the ability" Corrected lines 256.
Be consistent in whether use blackflies/blackfly vs black flies/black fly [I prefer the former]. Corrected lines 25, 68, 91 and throughout.
I'm not really sure what the term "reinforced treatment” corrected see lines 537, or "assimilate to" Modified. See lines 246-247 or "sensitisation of the people" Sensitisation has been replaced with awareness. See lines 303-305 for definition of awareness. CDDs has already been defined previously. Removed, line 256.

2nd paragraph conflates identification of additional communities with the challenges of reaching people in settlements; please clean this up. Thank you. We now revised. See line 507.

p27
first paragraph -- I don't think the authors mean "quality of treatment"? Poor coverage perhaps? Thank you. This is elaborated upon in paragraph line 520.

"Better quality has been achieved in terms of" -- not very well worded; "Coverage estimates have been improved because of..."? Or just "the Action Plan improved accuracy in drug..."? Thank you. This now corrected and updated. Line 520.

"This improvement will be impactful..." also needs some copyediting of this sentence please. Thank you. This now corrected and updated. Lines 528.

Figures:
Figure 1-- Recommend adding an inset map of Ghana indicating the relevant area under consideration for context. The legend is very confusing. Why is parasitology a pie chart? Is this the proportion of people who were skin snip mf+ve? Why is parasitology a pie chart while serology is a colored range? Is the larvae found supposed to indicate mf or iL3 larvae, perhaps blackfly larvae?

Thank you for your detailed feedback. We have now included map of Ghana and clarified in Figure 1 caption that the focus area is within a 10 km radius. This map has been published and we have modified clear for better context.

Regarding the legend:

- We acknowledge the confusion and have revised the legend to better distinguish between parasitology and serology indicators.
- The parasitology pie chart represents the proportion of individuals who tested positive for microfilariae (mf+) via skin snip.
- The serology data are presented as a color gradient to reflect antibody prevalence across the area.
- The larvae symbols refer to infective L3 larvae found in blackflies, and we have updated the figure legend to clarify this distinction.

Figure 2-- Typos in "analyse data" set. Corrected: Figure 2.

"gaps in MDA coverage, map areas with persistent infection, map areas with inadequate...". Shouldn't identifying barriers, solutions, and opportunities to participate be a separate step from data analysis, given that this is interpreting the data? Isn't this part meant to be done in consultation with key community people?

Thank you very much. We have moved these activities to the Action Plan development step. Figure 2.

Title: Develop Action-- add "Plan". Done; Figure 2.

Note that "enhance community engagement" assumes that this is a problem a priori, while it might not be in all areas and thus in some areas would be identified as a solution in the Action Plan.

Thank you for your observation. We agree that the phrase "enhance community engagement" may suggest a pre-existing problem, which may not apply in all areas. To better reflect this nuance, we have removed the word "enhanced." Figure 2.

Typo "ensure continued community engagement". Corrected; Figure 2.

Figure 3--legend indicates that this is a "transmission focus", but need to indicate in text the data that suggest that this 10km radius is a transmission focus and not an arbitrary delimitation.

Thank you for this important observation. We have addressed this in the Background and method (setting) sections, where we explain that the 10 km radius was defined based on parasitological, serological, and entomological data. See lines 99-107. Also see updated Figure 3 legend

Copyright notice needs to be part of sentence that precedes it so that it doesn't mis-label the figure itself as (c) Esri.

Copy right noticed has been removed in line with journal guidelines/requirements. Figure 3.

Reviewer #2: (No Response)

**Summary and General Comments**


Use this section to provide overall comments, discuss strengths/weaknesses of the study, novelty, significance, general execution and scholarship. You may also include additional comments for the author, including concerns about dual publication, research ethics, or publication ethics. If requesting major revision, please articulate the new experiments that are needed.

Reviewer #1: Onchocerciasis and other parasitic NTDs are close to elimination. However, hotspots of persistent transmission remain despite decades of drug distribution, and new solutions are needed. Nditanchou et al. have proposed an approach to addressing some of the problems that may be contributing to lack of successful MDA at the community level. This approach involves connecting community, health workers, and programme staff to identify what some of the problems are and to co-design a solution (an "action plan") that has clear accountability. Their argument is that such an approach will lead to improved MDA coverage.

I enjoyed the manuscript and would like to see the study published. However, because the study takes place in a small area in Ghana, the study itself is not what makes this paper interesting. I recommend that the authors strengthen their framing of this in the Introduction and Discussion more explicitly as an approach to solve a problem, using Kwanware-Ottou as a test for broader roll-out. The way it is currently written, the focus is more on the results from Kwanware-Ottou.

Thank you for your positive feedback and helpful suggestion. We have now revised the Introduction and Discussion to more clearly position the study as a demonstration of an approach to addressing transmission and CDTI performance issue with Kwanware-Ottou serving as a test case. This framing emphasizes the broader applicability and potential for scaling the approach beyond the immediate study area, as you recommended. See lines 108-112; 474-484; 573-602.

I further think that there needs to be more clarity in the Discussion with regards to the limitations are in their approach, and how they would recommend other programmes proceed in implementing this type of approach given their experiences (e.g., geographic scale, metrics for evaluating success, time frame). This would make the paper far more applicable to the broader NTD audience that the journal targets.

Thank you for this helpful suggestion. We have revised the Discussion to clarify the limitations of our approach and included practical recommendations for other programmes—addressing geographic scale, success metrics, and time frames—to enhance relevance for the broader NTD audience. Lines 550-602.

There is significant value in this study--for example, the description of how activities were designed to "develop capacity at every level" was great! Appendix 3 represented a clear summary and was very interesting. The amount of coordination and work that went into the project is to be commended.

Thank you for your thoughtful feedback.

Reviewer #2: I do not think this paper can be published in its current form. Leaving aside what I think are some serious inconsistnencies in the data that are reported, there is simply insufficient evidence presented to support most of the claims and conclusions beyond those that relate to better defining the location and size of the target population. Nothing can be concluded about the likely causes of the apparently low historic coverage (although the coverage information presented is of doubtful validity) and there are no data presented to support either the action plan described nor the claim that coverage improved as a result. Substantial revision is required if this paper is to be published.

Thank you for your feedback. While we respectfully disagree that the coverage data lacks validity or that the action plan lacks a solid basis, we acknowledge that the evidence of improvement could have been presented more strongly. We have now revised the manuscript to include programme-reported coverage data before and after implementation, providing clearer and stronger support for the impact of the intervention. See lines 440-459; 520-530

To be more positive, I do agree that it is extremely important to investigate the causes for persistent hotspots of transmission. It is clear that such hotspots are emerging in many onchocerciasis endemic settings throughout Africa, particularly where annual MDA is the sole tool employed. Low therapeutic coverage may be a cause in some of these, but (a) a much more rigorously quantiative approach than that taken here is required to demonstrate conclusively that this is indeed the likely cause of persistent transmission

Thank you for this thoughtful observation. We fully agree. As stated in our introduction, this should indeed be the first step, given the demonstrated effectiveness of MDA. Regardless of the underlying cause of persistent transmission, if there is no drug resistance, MDA will remain central to the response. Please refer to lines 99–127 for further context. And lines 573-602. Nevertheless, our previous work has investigated deeper. See reference 11.

and (b) more rigorous analysis of the outcome of remedial actions is likewise required that the entirely qualitative claim of success that is made in this paper. My strong recommendation to the authors is to move away from anecdotal, qualtiative, subjective reporting to the use of tools such as Nviva to summarise and analyse qualitative data in a more objective, quantitative fashion.

Thank you for your feedback. We’ve added structured programme-reported coverage data post-intervention, alongside pre-intervention survey data, to support improved data quality and coverage. See lines 441-459.

While a formal coverage evaluation would offer a direct comparison, the programme’s overhaul—including better registration, outreach, and supervision—supports the reliability of post-intervention data. See lines 202-204; 520-524. Given this, we do not see added value in NVivo visual analysis.

As noted earlier, NVivo visuals are not standard reporting practice in qualitative research.

Confer:

1. Allsop DB, Chelladurai JM, Kimball ER, Marks LD, Hendricks JJ. Qualitative methods with Nvivo software: A practical guide for analyzing qualitative data. Psych. 2022 Mar 22;4(2):142-59. [**https://doi.org/10.3390/psych4020013**](https://doi.org/10.3390/psych4020013)
2. Paulus T, Woods M, Atkins DP, Macklin R. The discourse of QDAS: Reporting practices of ATLAS. ti and NVivo users with implications for best practices. International Journal of Social Research Methodology. 2017 Jan 2;20(1):35-47. <https://doi.org/10.1080/13645579.2015.1102454>
